# The isoquinoline PRL-295 increases the thermostability of Keap1 and disrupts its interaction with Nrf2

**DOI:** 10.1016/j.isci.2021.103703

**Published:** 2021-12-27

**Authors:** Sharadha Dayalan Naidu, Takafumi Suzuki, Dina Dikovskaya, Elena V. Knatko, Maureen Higgins, Miu Sato, Miroslav Novak, José A. Villegas, Terry W. Moore, Masayuki Yamamoto, Albena T. Dinkova-Kostova

**Affiliations:** 1 Division of Cellular Medicine, School of Medicine, Jacqui Wood Cancer Centre, Ninewells Hospital and Medical School, University of Dundee, James Arnott Drive, Dundee, Scotland DD1 9SY, UK; 2 Department of Medical Biochemistry, Tohoku University Graduate School of Medicine, Sendai, Japan; 3 Department of Pharmaceutical Sciences, College of Pharmacy, University of Illinois Cancer Center, University of Illinois at Chicago, Chicago, IL 60607, USA; 4 Department of Pharmacology and Molecular Sciences and Department of Medicine, Johns Hopkins University School of Medicine, Baltimore, MD 21205, USA

**Keywords:** Biological sciences, Biochemistry, Molecular interaction

## Abstract

Transcription factor Nrf2 and its negative regulator Keap1 orchestrate a cytoprotective response against oxidative, metabolic, and inflammatory stress. Keap1 is a drug target, with several small molecules in drug development. Here, we show that the isoquinoline PRL-295 increased Keap1 thermostability in lysates from cells expressing fluorescently tagged Keap1. The thermostability of endogenous Keap1 also increased in intact cells and murine liver following PRL-295 treatment. Fluorescence Lifetime Imaging–Förster Resonance Energy Transfer (FLIM-FRET) experiments in cells co-expressing sfGFP-Nrf2 and Keap1-mCherry further showed that PRL-295 prolonged the donor fluorescence lifetime, indicating disruption of the Keap1-Nrf2 protein complex. Orally administered PRL-295 to mice activated the Nrf2transcriptional target NAD(P)H:quinone oxidoreductase 1 (NQO1) in liver and decreased the levels of plasma alanine aminotransferase and aspartate aminotransferase upon acetaminophen-induced hepatic injury. Thus, PRL-295 engages the Keap1 protein target in cells and *in vivo*, disrupting its interaction with Nrf2, leading to activation of Nrf2-dependent transcription and hepatocellular protection.

## Highlights

• PRL-295 increases the thermostability of Keap1 in cells and in the murine liver
• PRL-295 disrupts the Keap1-Nrf2 protein complex in single live cells
• Orally administered PRL-295 activates the Nrf2 transcriptional target NQO1 in murine liver
• Orally administered PRL-295 to mice decreases acetaminophen-induced hepatic injury

## Graphical abstract

**Figure:**
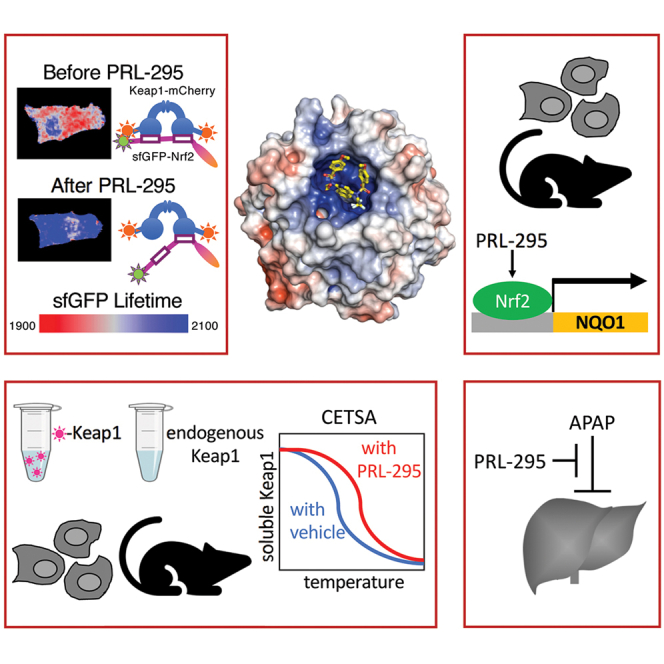

## Introduction

The transcription factor nuclear factor erythroid 2 p45-related factor 2 (Nrf2, encoded by *NFE2L2*) and its main negative regulator, the Kelch-like ECH associated protein 1 (Keap1), orchestrate the coordinated expression of large networks of proteins, allowing cellular and organismal adaptation to oxidative, electrophilic, metabolic, and inflammatory stress (Hayes and Dinkova-Kostova, 2014; Yamamoto et al., 2018). Under homeostatic conditions, Keap1 serves as a substrate adaptor for a Cullin3-based RING E3 ubiquitin ligase, which continuously targets Nrf2 for ubiquitination and proteasomal degradation. This process can be disrupted by numerous small molecules, which target Keap1 either by covalently modifying its sensor cysteines or by binding to it non-covalently and inhibiting its protein–protein interactions with Nrf2 (Cuadrado et al., 2019). Genetic or pharmacological activation of Nrf2 results in a broad cytoprotection in a plethora of animal models of acute and chronic human diseases, including diseases of the liver, the brain, the lungs, the eyes, the heart, the kidneys, and the skin (Liby and Sporn, 2012), and has provided a strong rationale for clinical translation. Indeed, clinical trials with the electrophilic Nrf2 inducers sulforaphane, dimethyl fumarate, bardoxolone methyl, and oltipraz have demonstrated beneficial effects in humans, and some are promising protective agents against unavoidable exposures to environmental pollutants (Yagishita et al., 2019, 2020). The Keap1/Nrf2 protein complex is now a recognized drug target, several Nrf2 activators are in advanced clinical trials, including in patients with COVID-19, and dimethyl fumarate is used in clinical practice for the treatment of psoriasis (under the brand name Skilarence) and relapsing forms of multiple sclerosis (brand name Tecfidera) (Atanasov et al., 2021; Cuadrado et al., 2019, 2020).

The remarkable preclinical efficacy of electrophilic Nrf2 inducers and the successful clinical implementation of dimethyl fumarate have inspired the design and/or identification of non-electrophilic Keap1-Nrf2 protein–protein interaction inhibitors, which are expected to have greater selectivity, metabolic stability, and safety profiles. Consequently, a number of peptide and small molecule non-electrophilic compounds that directly and reversibly inhibit the interactions between Keap1 and Nrf2 have been described; the latter belong to distinct chemical classes, including isoquinoline, naphthalene sulfonamide, benzo[*g*]indole, oxadiazole, and triazole classes (Abed et al., 2015; Davies et al., 2016; Jain et al., 2015; Jiang et al., 2014; Mou et al., 2020; Richardson et al., 2018; Wells, 2015). In addition, a recently developed highly automated open-source platform (termed VirtualFlow) for structure-based virtual screening was employed to screen more than 1 billion compounds, leading to the identification of several structurally diverse small molecules that bind to Keap1 with affinities in the low-to sub-micromolar range (Gorgulla et al., 2020).

**Figure 1:**
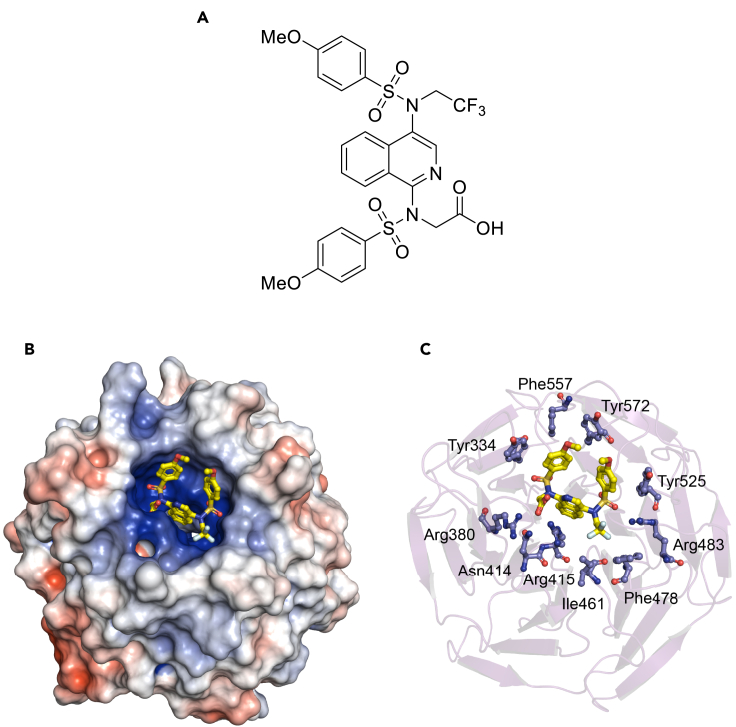
The Keap1-Nrf2 protein–protein interaction inhibitor PRL-295 targets the Kelch domain of Keap1 (A) Chemical structure of PRL-295. (B and C) Pymol renderings of PRL-295 (yellow and atom colors) bound to the Kelch domain of Keap1 shown as either an electrostatic potential map (B) or as a ribbon diagram with interacting residues labeled (C). PDB ID: 6UF0.

One recently reported Keap1-Nrf2 protein–protein interaction inhibitor is the 2,2,2-trifluoroethyl monoacid isoquinoline PRL-295 (Figure 1A), which targets the Kelch domain of Keap1, the site of interaction with Nrf2 (Lazzara et al., 2020). The electrostatic potential map of the Kelch domain of Keap1 calculated from the co-crystal structure with PRL-295 (PDB: 6UF0) using the Adaptive Poisson-Boltzmann Solver (APBS) software (Jurrus et al., 2018) shows a highly positively charged binding pocket, with the carboxylate group of PRL-295 in close contact with Arg380, Asn414, and Arg415, and the sulfonamide oxygens within hydrogen bond distance of Ser508, Ser555, and Ser602 oxygens (<3.5 Å, Figures 1B and 1C). Notably, Keap1 is a homodimeric protein, which binds through its Kelch domains to one molecule of Nrf2 via two sites located in the N-terminal Neh2 domain of Nrf2, a low-affinity “DLG” motif, and a high-affinity “ETGE” motif (McMahon et al., 2006; Tong et al., 2006), whereby acidic residues in these two motifs interact electrostatically with a triad of arginine residues (Arg380, Arg415, and Arg483) in each of the Kelch domains of the Keap1 homodimer (Tong et al., 2007). Crucially, binding to both motifs is required for Nrf2 ubiquitination and subsequent degradation. Fluorescence Lifetime Imaging - Förster Resonance Energy Transfer (FLIM-FRET) experiments in single live cells ectopically co-expressing EGFP-Nrf2 and Keap1-mCherry fusion proteins suggested that binding occurs sequentially, first through the high-affinity “ETGE” motif, giving rise to an “open” conformation of the EGFP-Nrf2: Keap1-mCherry protein complex, followed by binding through the low-affinity “DLG” motif to form the fully bound “closed” conformation (Baird et al., 2013). The use of this experimental system further showed that electrophiles promote the formation of the “closed” conformation of the EGFP-Nrf2: Keap1-mCherry protein complex, suggesting that the newly synthesized Nrf2 accumulates due to insufficient availability of free Keap1 (Baird et al., 2013). By contrast, exposure to a non-electrophilic Keap1-Nrf2 protein–protein interaction inhibitor of the triazole chemical class favored formation of the “open” conformation (Bertrand et al., 2015). In agreement, recent nuclear magnetic resonance (NMR) spectroscopy titration experiments with recombinantly produced full-length Keap1 homodimeric protein and stable isotope [^13^C and ^15^N]-labeled Neh2 domain of Nrf2 protein revealed that PRL-295 disrupts the binding of Keap1 to the “DLG” motif preferentially to the binding of Keap1 to the “ETGE” motif of Neh2 (Horie et al., 2021). In this contribution, we show that PRL-295 binds to Keap1 in the cellular environment, disrupting its interaction with Nrf2, which leads to enhanced gene expression of the classical Nrf2 target NAD(P)H:quinone oxidoreductase 1 (NQO1) in cells and *in vivo* in the mouse liver.

## Results

### PRL-295 increases the thermostability of Keap1 in cell lysates and in live cells

**Figure 2:**
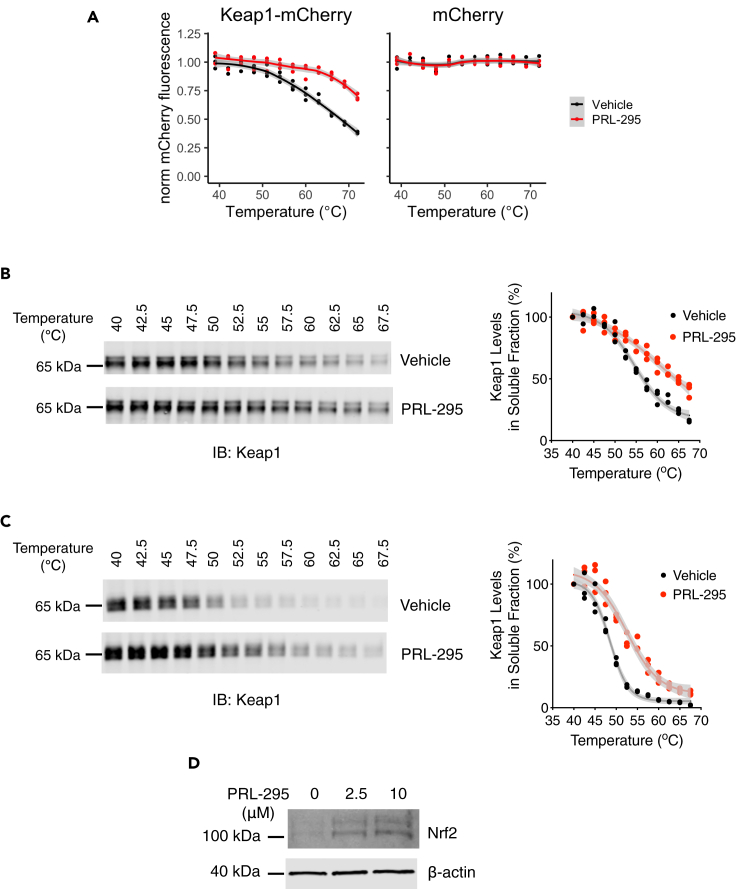
PRL-295 increases the thermostability of Keap1 in cell lysates and in live cells (A) Keap1-mCherry is stabilized by PRL-295 in cell lysates. Temperature-induced aggregation curves of Keap1-mCherry (left) or mCherry (right) were obtained following a 1-h pre-incubation of Keap1-mCherry- or mCherry-expressing U2OS cells lysates with 15 μM PRL-295 (red) or vehicle (0.05% DMSO, black), by mCherry fluorescence intensity in soluble fractions of lysates heated to 39°C–72°C, normalized to that of soluble fraction of DMSO-treated lysate heated to 39°C. Triplicate samples were measured for each point, and the temperature-induced aggregation curves were visualized by fitting local polynomial regression lines. Note that PRL-295 induces a thermal shift in Keap1-mCherry but not free mCherry-expressing lysates. Shown are mean values ± 1 standard error of means (SEM). (B and C) Endogenous Keap1 is stabilized by PRL-295 in cell lysates and intact cells. (B) Temperature-induced aggregation curves of Keap1 obtained by densitometric analysis of immunoblots of soluble fractions of HL-60 cell lysates, which had been heated to 40°C–67.5°C following a 1-h pre-incubation with 30 μM PRL-295 or vehicle (0.1% DMSO). Shown are individual datapoints for three replicates for each condition. (C) Temperature-induced aggregation curves of Keap1 obtained by densitometric analysis of immunoblots of soluble fractions of lysates prepared from HL-60 cells, which had been heated to 40°C–67.5°C following a 3-h treatment with 10 μM PRL-295 or vehicle (0.1% DMSO). Shown are individual datapoints for three replicates for each condition. (D) Protein levels of Nrf2 in differentiated HL-60 cells, which had been treated with PRL-295 (2.5 μM or 10 μM) or vehicle (0.1% DMSO) for 3 h.

We assessed the interaction between PRL-295 and Keap1 ectopically expressed as a fluorescently tagged protein that was genomically integrated into human osteosarcoma U2OS cells, using a modified cellular thermal shift assay (CETSA) (Jafari et al., 2014) that we developed for this purpose. U2OS cells were selected based on their high transfection efficiency and suitability for stable ectopic gene expression in mammalian cells. In this assay, the thermostability of Keap1-mCherry protein in lysates obtained from the above cells was measured by the fluorescence of soluble fraction that remains after removal of heat-induced aggregates, over a wide range of temperatures. A one-hour preincubation of the lysates with 15 μM PRL-295 increased the thermostability of Keap1-mCherry compared to vehicle (0.05% DMSO) treatment (Figure 2A, left panel), indicating that there is an interaction between the fluorescent protein and the compound. Importantly, the thermostability of mCherry in lysates from control U2OS cells that ectopically express mCherry alone was not affected by PRL-295 in an analogous setting (Figure 2A, right panel), confirming that PRL-295 targets the Keap1 portion of the Keap1-mCherry fusion protein.

To determine whether PRL-295 binds to endogenous Keap1, we employed an immunoblot-based CETSA. For these experiments we used human promyelocytic leukemia HL-60 cells, which grow in suspension and thus provide a much more robust system than adherent cells for collecting cell pellets for treatment with compounds without the need for trypsinization or mechanical scraping. Cell lysates were prepared and treated with PRL-295 (30 μM) for 1 h at 37°C, following which aliquots of the cell lysates were subjected to a temperature gradient. The soluble and insoluble protein fractions were then separated by centrifugation, and the levels of Keap1 in the soluble fraction were determined by immunoblotting. This analysis showed that the thermal stability of Keap1 was increased by the PRL-295 treatment (Figure 2B), demonstrating that the compound binds to endogenous human Keap1. Of note, the untagged endogenous Keap1 was less thermostable than the Keap1-mCherry fusion, consistent with previously described stabilization effect of mCherry (Mestrom et al., 2019). Importantly, the effect of PRL-295 on Keap1 was clearly evident in both systems.

To further confirm that PRL-295 crosses the cell membrane and subsequently binds to Keap1 in the cellular environment, we performed the CETSA in intact cells. Live HL-60 cells were treated with PRL-295 (10 μM) or vehicle for 3 h at 37°C, and treated intact cells were subjected to the same temperature gradient. The cells were subsequently lysed, the lysates cleared of the insoluble aggregates, and the levels of soluble Keap1 detected and quantified using Western blot analysis. Similar to the treatment of cell lysates, exposure of intact HL-60 cells to PRL-295 increased the thermal stability of Keap1 (Figure 2C). As expected, the levels of Nrf2 increased following a 3-htreatment with PRL-295 in a concentration-dependent manner (Figure 2D). These data establish that PRL-295 binds to Keap1 and leads to Nrf2 accumulation in intact live cells.

### PRL-295 disrupts the interaction between Keap1 and Nrf2 in single live cells

**Figure 3:**
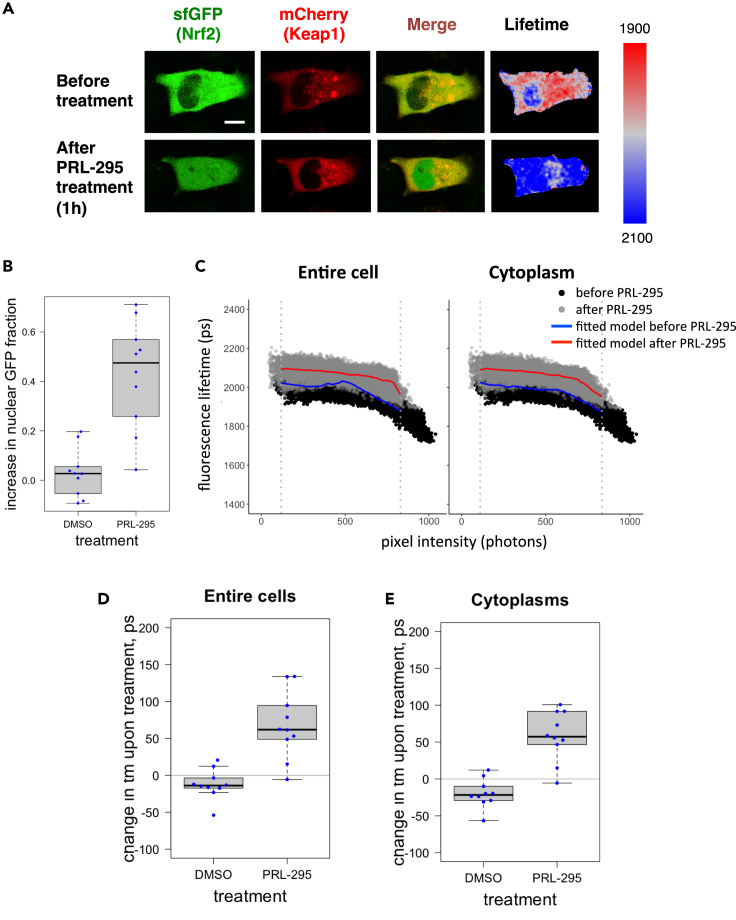
PRL-295 disrupts the interaction between Keap1 and Nrf2 in single live cells (A) Representative images of a cell co-expressing sfGFP-Nrf2 and Keap1-mCherry before (top row) and after (bottom row) a 1-h treatment with PRL-295 (50 μM) at 37°C. Single optical sections (columns 1–3) were captured in the green channel suitable for sfGFP and the red channel suitable for mCherry using confocal microscopy. The first column shows sfGFP, the second column shows mCherry, and the third column shows their overlay. The fourth column shows the fluorescence lifetime (tm) of sfGFP, which was determined by one-exponential decay fitting of the photon arrival times, measured in each pixel using FLIM (spectrally color-coded between 1900 and 2100 ps, as indicated on the side bar). Scale bar = 10 μm. (B) Quantification of the increase in nuclear sfGFP fraction following a 1-h treatment with PRL-295 (50 μM) or vehicle (0.1% DMSO) at 37°C. Each dot represents the increase of sfGFP in the nucleus of an individual cell. (C)Fluorescence lifetime of each pixel of the FLIM image (exemplified in the fourth column in A.) of a cell before (black) or after (gray) PRL-295 treatment is plotted against its intensity (photon count). The solid lines depict local polynomial regressions (“loess”) models fitted to the FLIM data of the cell before (blue) or after (red) treatment, either in the entire cellular area (left) or within the cytoplasmic regions (right). The dashed vertical lines outline the overlapping fluorescence intensity range between the two measurements (after removing 0.5% of brightest and 0.5% of dimmest of pixels) that excludes the non-comparable data. Note that the difference in fluorescence lifetime between pre- and post-treatment samples is apparent throughout the entire range of intensities, evident by the treatment-induced vertical shift of the fitted model curve. (D and E) Changes in tm values in entire cells (D) and cytoplasmic areas of imaged cells (E) following a 1-h treatment with PRL-295 (50 μM) or vehicle (0.1% DMSO) at 37°C, calculated as the mean treatment-induced displacement of the polynomial regression fitted to the distribution of fluorescence lifetimes among pixels of different intensities, within the overlapping intensity range as shown in (C) Each dot represents the change in tm in either the entire cell or the cytoplasm of an individual cell. The boxplots in B, D, and E show the distribution of values within each group of identically transfected cells: the box outlines the range between the 25th and 75th percentiles, the thick line within the box marks the median, and the whiskers show minimum and maximum values that extend from the box by no more than 1.5 times interquartile range (the distance between the 25th and 75th percentiles).

Fluorescence lifetime changes can be indicative of alterations in the conformation of complexes of fluorescently tagged proteins. Using Fluorescence Lifetime Imaging (FLIM), we have previously shown that the fluorescence lifetime of EGFP-Nrf2 is shorter in cells co-expressing Keap1-mCherry compared with cells co-expressing EGFP-Nrf2 and free mCherry (Baird et al., 2013). This is due to Förster Resonance Energy Transfer (FRET) between the EGFP donor and the mCherry acceptor consequent to Nrf2 binding to Keap1, which brings the fluorophores in close proximity. To test the consequences of binding of PRL-295 to Keap1 on the conformation of the Keap1-Nrf2 protein complex in live cells, we used a recently developed methodology based on FLIM-FRET (Dikovskaya et al., 2019; Dikovskaya and Dinkova-Kostova, 2020). Similar to the principle of our earlier method, the proximity of ectopically expressed fluorescently tagged Nrf2 (sfGFP-Nrf2) and Keap1 (Keap1-mCherry) within the complex is assessed by the level of FRET between these molecules, which leads to shortening of the fluorescence lifetime of the FRET donor, superfolder GFP (sfGFP). Importantly, as its name suggests, sfGFP has very fast maturation kinetics, matching the short half-life of endogenous Nrf2. For these experiments, we used HeLa cells because they have high transfection efficiency and morphology that is well suited for imaging experiments. We co-expressed sfGFP-Nrf2 and Keap1-mCherry, treated the cells with PRL-295 (50 μM) for 1 h at 37°C, and quantified the changes in fluorescence lifetime of sfGFP-Nrf2. Treatment with PRL-295 caused nuclear accumulation of sfGFP-Nrf2 (Figures 3A and 3B), consistent with Nrf2 stabilization and nuclear translocation. The fluorescence lifetime of sfGFP-Nrf2 was increased by the compound treatment (Figures 3A and 3C), suggesting reduction in FRET between the sfGFP and mCherry fluorophores, which corresponds to a decrease in the interaction between Keap1-mCherry and sfGFP-Nrf2. The treatment-induced increase in fluorescence lifetime was evident for all levels of sfGFP-Nrf2 within the cellular area, apparent from direct comparison of pre- and post-treatment fluorescence lifetime data plotted against their pixel intensities (Figure 3C), indicating that it was not an artifact of altered fluorescence intensity. Quantification of intensity-independent changes in fluorescence lifetime using previously developed algorithm (Dikovskaya and Dinkova-Kostova, 2020) confirmed that the increase in fluorescence lifetime was specific to PRL-295 and did not occur with the vehicle (0.1% DMSO) treatment (Figures 3D and 3E). The difference between the effects of DMSO and PRL-295 was highly statistically significant (p values 0.00023 for the entire cells and 1.85e-05 for cytoplasmic areas). Taken together, the results from these experiments demonstrate that PRL-295 binds to Keap1 and disrupts its interaction with Nrf2, leading to Nrf2 nuclear accumulation.

### PRL-295 induces the Nrf2-target enzyme NQO1 in cells and in the murine liver

**Figure 4:**
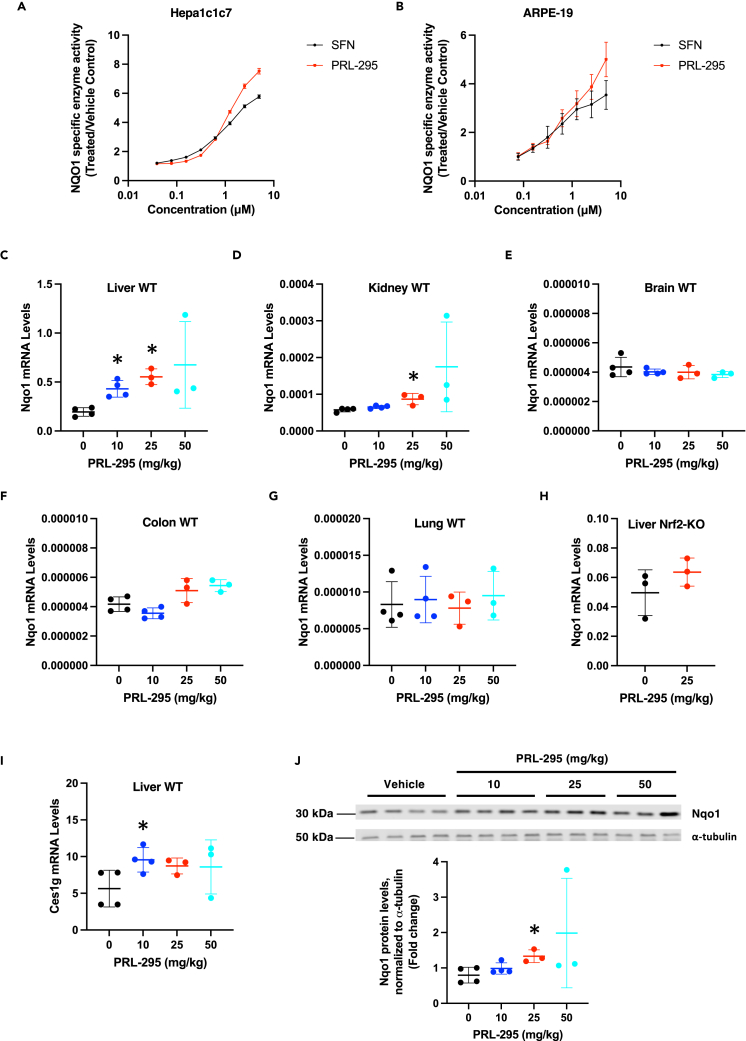
PRL-295 induces the Nrf2-target enzyme NQO1 in cells and in the murine liver (A and B) Specific enzyme activity of NQO1 after a 48-h treatment with PRL-295 or sulforaphane (SFN) in Hepa1c1c7 (A) and ARPE-19 (B) cells growing in 8 replicate wells. The data are expressed as the mean values ± standard error of means (SEM) of the ratio of the NQO1 specific enzyme activity in lysates from PRL-295-treated cells over control (0.1% DMSO vehicle-treated) cells. (C–G) mRNA levels for NQO1 in livers (C), kidneys (D), brains (E), colons (F), and lungs (G) of male C57BL/6 WT mice (n = 3–4) that had been treated with vehicle (5% DMSO in corn oil) or PRL-295 at the indicated doses, *per os*, 4 times, 24 h apart; tissues were harvested 3 h after the last dose. (H) mRNA levels for NQO1 in livers of male Nrf2-knockout (Nrf2-KO) C57BL/6 WT mice (n = 3) that had been treated with vehicle (5% DMSO in corn oil) or 25 mg/kg body weight PRL-295, *per os*, 4 times, 24 h apart; tissues were harvested 3 h after the last dose. (I) mRNA levels for Ces1g in livers of male C57BL/6 WT mice (n = 3–4) that had been treated with vehicle (5% DMSO in corn oil) or PRL-295 at the indicated doses, per os, 4 times, 24 h apart; tissues were harvested 3 h after the last dose. The data are presented as mean values ± 1 standard deviation (SD), and the datapoints for each individual animal are also shown. ∗p < 0.05. (J) Protein levels of NQO1 in livers of male C57BL/6 WT mice (n = 3–4) that had been treated with vehicle (5% DMSO in corn oil) or PRL-295 at the indicated doses, *per os*, 4 times, 24 h apart; tissues were harvested 3 h after the last dose. Shown are an immunoblot and its quantification for each animal.

To determine the inducer potency of PRL-295, we used a quantitative bioassay in murine hepatoma (Hepa1c1c7) cells, which measures the enzyme activity of the classical Nrf2 target, NAD(P)H:quinone oxidoreductase 1 (NQO1) as the readout (Fahey et al., 2004). The Hepa1c1c7 cell line represents one of the most sensitive and robustly responsive cell lines to Nrf2 activators, with a large dynamic range. Exposure to PRL-295 for 48 h led to a concentration-dependent induction of the specific enzyme activity of NQO1 with a concentration that doubles the NQO1 enzyme activity (CD) value of 200 nM (Figure 4A). The potency of the non-electrophilic PRL-295 was identical to the potency of the classical NQO1 inducer sulforaphane (SFN), which is an electrophile that activates Nrf2 by modifying cysteine sensors of Keap1 (Dayalan Naidu and Dinkova-Kostova, 2020), with C151 acting as the main sensor for sulforaphane (McMahon et al., 2010; Saito et al., 2015; Zhang and Hannink, 2003). Notably, the magnitude of induction by PRL-295 was greater than that by sulforaphane. As in mouse Hepa1c1c7 cells, treatment with PRL-295 led to a concentration-dependent induction of NQO1, with a potency similar to that of SFN, in human adult retinal pigment epithelial cells (ARPE-19) (Figure 4B), a diploid cell line with structural and functional properties that are very similar to retinal pigment epithelial cells *in vivo* (Dunn et al., 1996), with a potency comparable to that of SFN.

PRL-295 has been designed and shown to be metabolically stable (Lazzara et al., 2020), and we next examined its ability to induce NQO1 *in vivo*. Following oral administration, the hepatic mRNA levels for NQO1 increased in a dose-dependent manner by 2.2- (p = 0.0026) and 2.8-fold (p = 0.0006) when the animals were administered orally 10- or 25-mg/kg PRL-295, respectively, 4 times, 24 h apart (Figure 4C). Administration of the highest (50 mg/kg) dose led to an overall 3.5-fold upregulation, but this was not statistically significant (p = 0.0754), most likely due to the high variation (ranging from 2- to 5.9-fold) among the individual animals and the small (n = 3) sample size. A modest (1.5-fold) statistically significant (p = 0.0158) increase by the 25 mg/kg dose, and a trend for an increase by the highest (50 mg/kg) dose of PRL-295 was also observed in the kidneys of the animals (Figure 4D), although similar to the liver data, the latter was of high inter-individual variation (ranging from 1.4- to 5.2-fold) and did not reach statistical significance (p = 0.1043). By contrast, examination of the mRNA levels for NQO1 in the brains (Figure 4E), colons (Figure 4F) and lungs (Figure 4G) of the mice did not reveal any differences between the PRL-295-treated and vehicle-treated groups, suggesting that at the administered oral doses, Nrf2 activation was largely limited to liver and kidneys. Importantly, the increase in the mRNA levels for NQO1 was only observed in livers of wild-type mice, but not in their Nrf2-knockout counterparts (Figure 4H), showing that NQO1 induction by PRL-295 was a consequence of Nrf2 activation. This conclusion is further supported by the upregulation (by ∼1.5-fold) of the mRNA levels for carboxylesterase 1g (Ces1g), another Nrf2-transcriptional target (Knatko et al., 2020), in the livers of wild-type mice (Figure 4I). Immunoblotting analysis of liver homogenates prepared from each individual animal also showed the upregulation of the protein levels of NQO1 (Figure 4J), in close agreement with the mRNA data (Figure 4C).

### PRL-295 increases the thermostability of Keap1 in the murine liver

**Figure 5:**
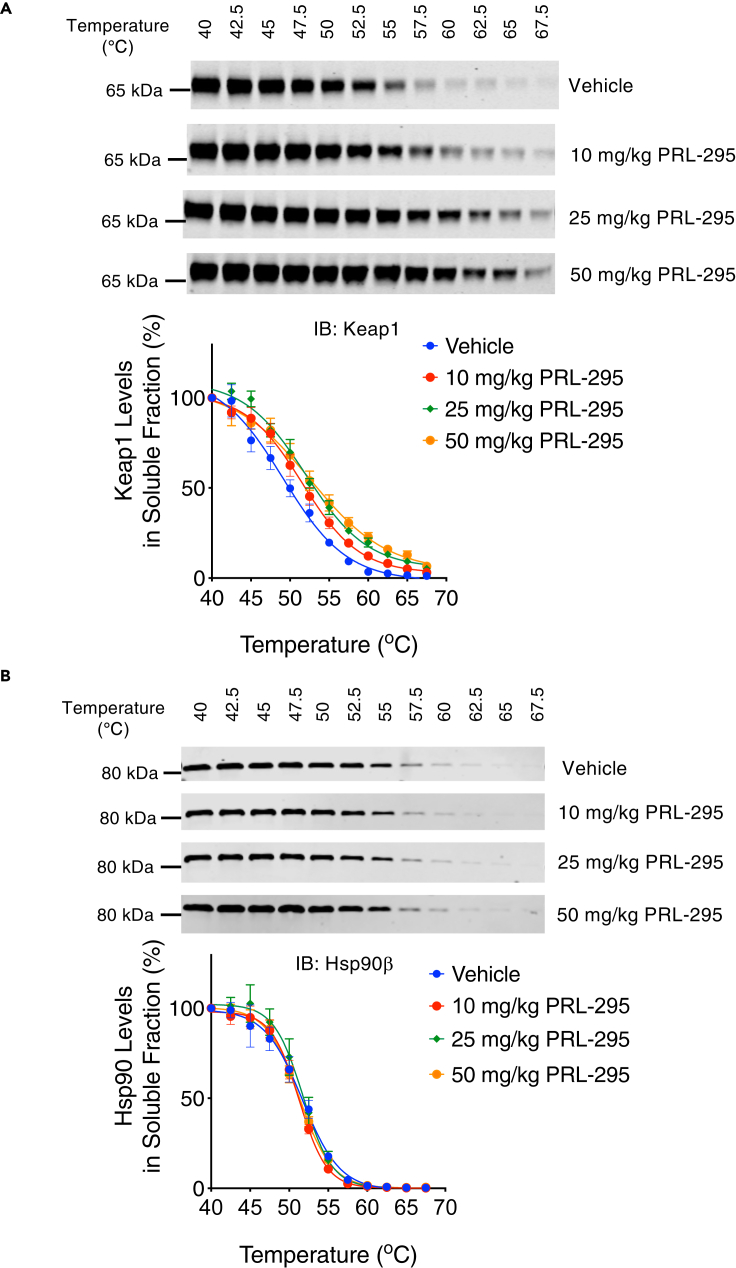
PRL-295 increases the thermostability of Keap1 in the murine liver (A and B) Temperature-induced aggregation curves of Keap1 (A) and Hsp90β (B) obtained by densitometric analysis of immunoblots of soluble fractions of liver homogenates heated to 40°C–67.5°C. The mice had been treated with vehicle (5% DMSO in corn oil, n = 4), 10 mg/kg body weight PRL-295 (n = 4), 25 mg/kg body weight PRL-295 (n = 3), or 50 mg/kg body weight PRL-295 (n = 3), *per os*, 4 times, 24 h apart; harvest was 3 h after the last dose. The data are represented as mean values ± 1 standard deviation (SD). Shown are representative immunoblots for one animal from each treatment group.

To confirm PRL-295 binding to Keap1 *in vivo*, we examined the thermostability of Keap1 in liver tissues from mice that were either treated with a vehicle or PRL-295 (10, 25 or 50 mg/kg, 4 times, 24 h apart). Livers were harvested 3 h after the last dose, liver homogenates were prepared and subjected to the same temperature gradient as in the above experiments with cells and cell lysates, and the protein levels of Keap1 in the soluble fractions were detected by immunoblotting. The analysis of the temperature-induced aggregation curves showed that, similar to the treatment of cell lysates and live cells, oral administration of PRL-295 to mice dose-dependently increased the thermal stability of Keap1 in the liver (Figure 5A). In contrast, the PRL-295 treatment did not alter the temperature-induced aggregation curves for Hsp90β, one of the most abundant cellular proteins (Figure 5B). Together, these data establish that PRL-295 binds to Keap1 *in vivo* and activates Nrf2 in the murine liver.

### The activation of Nrf2 by PRL-295 protects against acetaminophen-induced hepatotoxicity in mice

**Figure 6:**
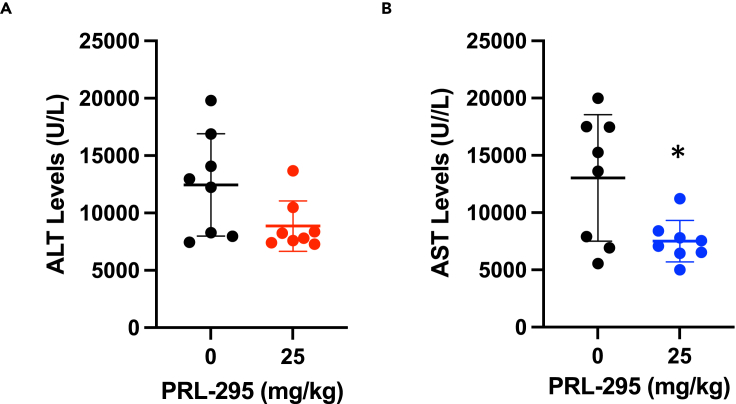
Nrf2 activation by PRL-295 protects against acetaminophen-induced hepatotoxicity (A and B)Levels of ALT (A) and AST (B) in blood of 8-week-old male mice (n = 8) that had been pre-treated with vehicle of PRL-295 (25 mg/kg body weight, *per os*), 3 times, 24 h apart, treated with APAP (300 mg/kg body weight, *i.p.*) after a further 24 h, and euthanized 24 h following APAP treatment. The data are presented as mean values ± 1 standard deviation (SD), and the datapoints for each individual animal are also shown. ∗p < 0.05.

Compared to wild-type, mice bearing a hepatocyte-specific deletion of the *Keap1* gene are more resistant to the toxicity of acetaminophen (APAP) (Okawa et al., 2006). Although APAP is a commonly used nonprescription drug with effective analgesic and antipyretic properties, APAP overdose can lead to hepatocellular necrosis, and in the United States (US) alone, is responsible for 50,000 emergency room visits, 10,000 hospitalizations, and ∼500 deaths annually (Lee, 2017), and is the cause for the need of ∼20% of all liver transplantations (Yoon et al., 2016). Thus, we next asked whether inhibition of Keap1 by PRL-295 is protective in a mouse model of acute APAP hepatotoxicity. Male C57BL/6J mice (n = 10) were administered orally PRL-295 (25 mg/kg) or vehicle, once a day, over a period of 3 days, and following an overnight food withdrawal, treated with a single sub-lethal dose of APAP (300 mg/kg) 24 h after the last PRL-295 dose. The 25 mg/kg dose of PRL-295 was chosen, because it induced the most consistent statistically significant increase in the mRNA and protein levels of NQO1 from all doses tested (Figures 4C and 4J). Plasma alanine aminotransferase (ALT) and aspartate aminotransferase (AST) were measured as markers of APAP-induced hepatic injury at 24 h post APAP treatment. The ALT and the AST levels were highly variable among individual animals (Figures 6A and 6B). Despite this high inter-individual variability, the ALT and the AST levels were lower in the PRL-295-treated group compared to the vehicle-treated group (p = 0.0598 for ALT and p = 0.0176 for AST). This experiment suggests that similar to genetic deletion, pharmacological inhibition of Keap1 by PRL-295 has hepatoprotective effects.

## Discussion

The discovery of small molecule non-electrophilic compounds that directly bind to the Kelch domain of Keap1 and thereby inhibit its protein–protein interactions with Nrf2, leading to activation of Nrf2-driven cytoprotective responses, is an exciting area in drug development (Abed et al., 2015; Mou et al., 2020; Wells, 2015). Binding to the Kelch domain of Keap1 has been demonstrated for a number of non-electrophilic compounds from several chemical classes. Co-crystalization and NMR spectroscopy experiments with recombinant Keap1-Kelch protein and, in limited cases, recombinant full-length Keap1 protein, have provided detailed information on binding modes, facilitating further compound optimization. Our study extends this knowledge by investigating the binding of a non-electrophilic Nrf2 activator to Keap1 in cells and *in vivo*. Our results show that PRL-295 engages the Keap1 target in the cellular environment (Figures 2A–2C), and in the murine liver *in vivo* (Figure 5A), in good agreement with the published *in vitro* data (Horie et al., 2021; Lazzara et al., 2020). Consequent to binding to Keap1, PRL-295 disrupts its interaction with Nrf2 (Figure 3), causing an increase in the Nrf2 protein levels (Figure 2D), nuclear accumulation of the transcription factor (Figures 3A and 3B), and induction of its classical target gene NQO1 in mouse and human cells, and in the murine liver at the mRNA and the protein levels (Figure 4).

The cytoprotective activity of several non-electrophilic Nrf2 activators has been demonstrated in mouse models of ulcerative colitis (Lu et al., 2016), renal inflammation (Lu et al., 2019b), acute hepatotoxicity (Lu et al., 2019a), lipopolysaccharide (LPS)-induced cardiomyopathy (Jiang et al., 2018), and myocarditis (Meng et al., 2018). Because in our experiments the bioavailability of PRL-295 was largely limited to the liver (Figures 4C–4J), we chose to test the ability of this compound to protect against APAP-induced hepatotoxicity in a proof-of-principle study. Our quantitative analysis of blood ALT and AST levels showed protection by PRL-295 (Figures 6A and 6B), in agreement with the previously reported protective effects in this hepatic injury model by compounds that activate Nrf2 by this (i.e. inhibition of the Keap1-Nrf2 protein–protein interactions) (Lu et al., 2019a), as well as other distinct mechanisms, including inhibition of Keap1 by electrophiles (Noh et al., 2015), inhibition of Cullin 3 neddylation, an essential modification for the activation of all Cullin-RING E3 ligases (Zhou et al., 2021), hepatocyte-specific deletion of Keap1 (Okawa et al., 2006), and even via Keap1-independent Nrf2 activation (Lin et al., 2015; Palliyaguru et al., 2016). Finally, it is clear from our mouse experiments that further compound optimization is necessary to improve the bioavailability and *in vivo* efficacy of orally administrated PRL-295 beyond the liver.

## Limitations of the study

To examine the ability of PRL-295 to activate Nrf2 *in vivo*, in this study we used 8- to 9-month-old male mice, and have not examined the effect of the compound in female mice or in mice of different ages; the latter would be particularly important considering that the inducibility of Nrf2 decreases with age (Zhou et al., 2018). The comparison between WT and Nrf2-disrupted mice is a strong indicator that the ability of PRL-295 to induce NQO1 is dependent on Nrf2, and as discussed above, Nrf2 activation is known to protect against acetaminophen-induced hepatic injury. However, whether the hepatoprotective effect of PRL-295 in our experiments is mediated by Nrf2 activation has not been established. It was not possible to address this question by use of Nrf2-disrupted mice. This is because a large proportion of Nrf2-disrupted C57/BL6 mice displays a congenital intrahepatic shunt with accompanying changes in hepatic oxygen and protein expression gradients, decreased centrilobular expression of cytochrome P450 2e1 (Cyp2e1, the enzyme that mediates the bioactivation of acetaminophen), and diminished sensitivity to acetaminophen-induced hepatotoxicity (Skoko et al., 2014). It is also noteworthy that, in addition to Nrf2, Keap1 has other binding partners, but whether PRL-295 may affect their interaction with Keap1 has not been investigated.

## STAR★Methods

### Key resources table

**Table:** 

REAGENT or RESOURCE	SOURCE	IDENTIFIER
**Antibodies**		

Anti-Keap1 Antibody, clone 144	Sigma-Aldrich	Cat#MABS514
Nrf2 (D1Z9C) XP® Rabbit mAb	Cell Signaling Technology	Cat#12721, RRID: AB_2715528
Recombinant Anti-Hsp90 beta antibody [EPR16621]	Abcam	Cat#ab203085, RRID: AB_2800430
Mouse monoclonal anti-β-actin	Sigma-Aldrich	Cat#A5441, RRID: AB_476744
NQO1 (D6H3A) Rabbit mAb	Cell Signaling Technology	Cat#62262, RRID: AB_2799623
α-Tubulin (DM1A) Mouse mAb	Cell Signaling Technology	Cat#3873, RRID: AB_1904178

**Chemicals, peptides, and recombinant proteins**		

DMSO	Sigma-Aldrich	Cat#D2650-5X5ML
PRL-295	Terry W. Moore	Lazzara et al., (2020)
Doxycycline hyclate	Sigma	Cat#D9891

**Critical commercial assays**		

TaqMan™ Fast Advanced Master Mix	Thermo Fisher Scientific	Cat#4444557
Omniscript RT Kit	Qiagen	Cat#205113
ALT assay	FUJIFILM	DRI-CHEM 7000V
AST assay	FUJIFILM	DRI-CHEM 7000V

**Experimental models: Cell lines**		

HL-60	ATCC	Cat#CCL-240
Hepa1c1c7	ATCC	Cat#CRL-2026
ARPE-19	Ian Ganley	Montava-Garriga et al., (2020)
HeLa Ohio	Cell services-LIF, CRUK	HeLa-O; ECACC Cat#84121901
Doxycycline (Dox)-inducible Keap1-mCherry U2OS (Flp-In^TM^ T-Rex^TM^) cell line (KC-U2OS)	This paper	n/a
Doxycycline (Dox)-inducible mCherry U2OS (Flp-In^TM^ T-Rex^TM^) cell line (C-U2OS)	This paper	n/a

**Experimental models: Organisms/strains**		

Mus musculus C57BL/6 Wild type	In-house	Jackson Laboratory
Mus musculus C57BL/6 Nrf2-knockout	In-house	Itoh et al., (1997) and Higgins et al., (2009)

**Taqman**^**TM**^**Gene expression assays**		

*Nqo1* (mouse)	Applied Biosystems	Mm01253561_m1
*Ces1g* (mouse)	Applied Biosystems	Mm00491334_m1
*B2M* (mouse)	Applied Biosystems	Mm00437762_m1
*Hprt1* (mouse)	Applied Biosystems	Mm03024075_m1

**Recombinant DNA**		

pmCherry N1 plasmid	Clontech	Cat#632523
Keap-12fl-mCherry plasmid	Dina Dikovskaya	Dikovskaya et al. (2019)
sfGFP-Nrf2 plasmid	Dina Dikovskaya	Dikovskaya et al. (2019)
Plasmid DNA construct mCherry in pcDNA5/FRT/TO	This paper	n/a
Plasmid DNA construct Keap1-mCherry in pcDNA5/FRT/TO	This paper	n/a
pOG44	ThermoFisher Scientific	Cat#V600520

**Software and algorithms**		

Graphpad Prism 8.0 Software	GraphPad	https://www.graphpad.com/scientific-software/prism/
ImageStudio Lite Software	LI-COR	n/a
FLIM DAtaSet Tool (FLIMDAST)	Freely available at https://github.com/DinaDikovskaya/FLIMDAST	Dikovskaya and Dinkova-Kostova (2020)
Zen	Zeiss	n/a
SoftMax Pro. 7.4	Molecular Devices	n/a
SPCImage	Becker & Hickl	
SPCM	Becker & Hickl	n/a
ImageJ	Freely available at https://imagej.nih.gov/ij/	n/a
R	Freely available at https://www.r-project.org/	n/a
R studio	Freely available at https://www.rstudio.com/	n/a

**Other**		

Amersham™ Protran® Premium Western blotting membranes, 0.45 μm pore size	Sigma	Cat#GE10600003
Veriti™ 96-Well Fast Thermal Cycler	Thermo Fisher Scientific	Cat#4375305
XCell4 SureLock™Midi-Cell gel electrophoresis system	Thermo Fisher Scientific	Cat#WR0100
NuPAGE™ 4 to 12%, Bis-Tris, 1.0 mm, Midi Protein Gel, 26-well	Thermo Fisher Scientific	Cat#WG1403BOX
Criterion™Blotter With Plate Electrodes	Bio-Rad	Cat#1704070
SpectraMax M2 microplate reader	Molecular Devices	n/a
Odyssey CLx Infrared Imaging System	LI-COR	n/a
FluoroDish	World Precision Instruments	Cat#FD35-100
Zeiss LSM 710 confocal microscope with InTune laser with 40 MHz pulse frequency, equipped with environmental chamber for life-cell imaging	Zeiss	n/a
SPC-150 SimpleTau module for measuring TCSPC-FLIM, attached to above Zeiss LSM 710	Becker & Hicl	n/a

### Resource availability

#### Lead contact

Further information and requests for resources and reagents should be directed to the lead contact: Albena T. Dinkova-Kostova (a.dinkovakostova@dundee.ac.uk).

#### Materials availability

Materials are available from the lead contact upon reasonable request.

### Experimental model and subject details

#### Cell culture

All cell cultures were maintained in a humidified atmosphere of 95% air and 5% CO_2_ at 37°C, and were routinely tested to ensure that they were mycoplasma-free. Hepa1c1c7 cells were grown in α-Minimum Essential Medium (α-MEM) supplemented with 10% fetal bovine serum (FBS) that had been inactivated by heating for 90 min at 55°C with 1% (w/v) charcoal. HeLa cells and parental and stably transfected Flp-In^TM^ T-Rex^TM^ U2OS cells were cultured in Dulbecco's Modified Eagle's Medium (DMEM) supplemented with 10% heat-inactivated FBS. HL-60 cells were grown in suspension in Roswell Park Memorial Institute (RPMI) 1640 medium supplemented with 10% heat-inactivated FBS. ARPE-19 cells (kind gift from Ian Ganley, University of Dundee) (Montava-Garriga et al., 2020) were grown in a mixture of equal volumes of DMEM and Ham's F-12 medium supplemented with 10% heat- and charcoal-inactivated FBS.

#### Mouse models

All animal experiments were carried out in accordance with the regulations described in the U.K. Animals (Scientific Procedures) Act 1986, the Guidelines for Proper Conduct of Animal Experiments of the Ministry of Education, Culture, Sports, Science and Technology of Japan, and the Standards for Human Care and Use of Laboratory Animals of Tohoku University (Sendai, Japan). The study protocols were approved by the Animal Care Committees of the University of Dundee and Tohoku University, and mice were bred at the respective animal facilities of the two universities and maintained on a 12-hour light/12-hour dark cycle, 35% humidity, with free access to water and food (pelleted RM1 diet from SDS Ltd. or Labo MR stock from Nihon Nosan Kogyo Co., Tokyo, Japan). The experimental animals were wild-type and Nrf2-knockout C57/BL6, 8-9-month-old, male, for the experiment shown in Figure 5, and wild-type, 8-week-old, male, for the experiment shown in Figure 6. Of note, the Nrf2-knockout mice express transcriptionally inactive Nrf2-β-galactosidase fusion protein, and were originally generated on a mixed genetic background (Itoh et al., 1997), and later back-crossed onto the C57/BL6 genetic background (Higgins et al., 2009).

### Method details

#### Cloning and generation of Keap1-mCherry- and mCherry-expressing U2OS cell lines

To generate the Keap1-mCherry construct for integration into U2OS cells, Keap1-mCherry sequence was excised from Keap1-12fl-mCherry plasmid (Dikovskaya et al., 2019) via KpnI and a partial NotI digest and cloned into KpnI and NotI sites of pcDNA5/FRT/TO (Thermo Fisher Scientific). To obtain the control mCherry construct, the mCherry DNA sequence from pmCherry-N1 (Clontech) was cloned into pcDNA5/FRT/TO via KpnI and NotI sites. Both constructs were verified by sequencing. To integrate the constructs into FRT integration site of Flp-In^TM^ T-Rex^TM^ host U2OS cells (kind gift from Laureano de la Vega, University of Dundee), the cells were co-transfected with pOG44 Flp recombinase-expressing vector (Thermo Fisher Scientific, kind gift from Adrian Saurin, University of Dundee) and either pcDNA5/FRT/TO-Keap1-mCherry or pcDNA5/FRT/TO-mCherry, and selected with 250 μg/mL hygromycin B. The expression was confirmed by western blotting and by mCherry fluorescence in cells induced for two days with 0.5 μg/mL of doxycycline hyclate.

#### Keap1-cellular thermal shift assay (Keap1-CETSA)

For CETSA in cell lysates, HL-60 cells (∼3 × 10^7^) were pelleted by centrifugation at 300 x*g* for 4 min at room temperature (RT) and washed with phosphate-buffered saline (PBS) twice. The washed pellet containing the cells was resuspended in 3 mL of PBS containing 1X protease inhibitor cocktail (PIC) [PBS-PIC] (Roche) and immediately snap frozen in liquid N_2_. After the sample was frozen, it was thawed completely at 25°C. To obtain the cell lysate, four freeze-thaw cycles were performed and followed by centrifugation at 17,000 x*g* for 15 min at 4°C to remove cell debris. The supernatant (lysate) was transferred to a fresh 15 mL centrifuge tube. The lysate was split into two 1.5 mL microcentrifuge tubes, each containing 1.3 mL of lysate. The lysates were incubated with either vehicle (0.1% DMSO) or 30 μM of PRL-295 at 37°C for 1 hour. For CETSA in intact cells, HL-60 cells (∼3 × 10^7^) growing in RPMI 1640 medium supplemented with 10% heat-inactivated FBS, were incubated with either vehicle (0.1% DMSO) or 10 μM of PRL-295 at 37°C, 5% CO_2_, for 3 hours. Cells were then pelleted by centrifugation at 300 x*g* for 4 minutes at RT, and intact cells were prepared as above by washing in PBS twice and resuspending in 3 mL of PBS-PIC. For CETSA in mouse liver, 15 mg of powder derived from pulverized frozen mouse liver was homogenized in 1.5 mL of PBS-PIC in a 2.0 mL microcentrifuge tube, and the cell debris was removed by centrifugation at 17,000 x*g* for 30 minutes at 4°C. For each condition, 100 μL of lysate, intact cell suspension or tissue homogenate (90 μg) was aliquoted into 12 PCR tubes and subjected to a range of temperatures (40–67.5°C) for 3 minutes using the Veriflex blocks in the Veriti^TM^ 96-well thermal cycler (Thermo Fisher Scientific). The samples were cooled for a further 3 minutes at 25°C,and subsequently snap frozen. On the following day, the samples were thawed out in a 25°C water bath, transferred to their respective 1.5 mL microcentrifuge tubes, and subsequently subjected to centrifugation at 17,000 x*g* for 40 minutes at 4°C to pellet the insoluble fraction. Sixty μL of supernatant (soluble fraction) was transferred to a fresh microcentrifuge tube containing 20 μL of 4X LDS buffer (Thermo Fisher Scientific) and 8 μL of Sample Reducing Agent (Thermo Fisher Scientific), mixed by brief vortexing, and incubated at RT for 30 minutes. Following this, the samples were subjected to western blot analysis where 13.5 μL of each sample was loaded and run on 26-well 4–12% NuPAGE Bis-Tris gels (Thermo Fisher Scientific) with MOPS running buffer. The gels containing the samples are transferred onto 0.45-μm nitrocellulose membranes using the wet transfer system (BioRad). After completion of the transfer, the membrane was blocked in 5% (w/v) non-fat milk in PBS-0.1% Tween 20 (Milk-PBST) for 1 h before overnight (16 h) incubation at 4°C with the primary rat monoclonal Keap1 antibody (1:2,000 in Milk-PBST, MABS514, Millipore). Following primary antibody incubation, the blot was washed thrice in PBS-0.1% (v/v) Tween 20 (PBST) for 30 minutes before incubation with goat anti-rat 680/800 IRDye secondary antibody (1:20,000 in Milk-PBST) for 1 h at RT protected from light. The blots were washed thrice in PBST for 30 minutes before scanning using the Odyssey (LI-COR) imager, where the Keap1 band intensity for each condition was quantified and normalised to the 40°C sample intensity and plotted using GraphPad Prism 8.0 (GraphPad Software Inc.).

#### Keap1-glow CETSA

Flp-In^TM^ T-Rex^TM^ U2OS cells with stably integrated Keap1-mCherry or mCherry constructs were induced with 0.5 μg/mL of doxycycline hyclate for four days before collecting cells in PBS supplemented with protease inhibitor cocktail (Roche) and lysing by repeated freeze-thaw cycles. The lysates were cleared by centrifugation at 9,600 x*g*, and incubated with 15 μM PRL-295 or the corresponding volume of DMSO for 1 hour at 37°C. The treated lysates were heated for 3 minutes at the indicated range of temperatures, followed by a high-speed centrifugation at 17,000 x*g* at 4°C for 40 minutes to remove aggregated material. The fluorescence of supernatants was measured using Spectramax M2 plate reader, with 580 nm excitation, 590 nm cut-off and 615 nm emission wavelengths.

#### Live cell imaging

HeLa cells (2 × 10^5^) were seeded onto glass-bottom 3.5-cm cell culture dishes (FluoroDish, World Precision Instruments). On the next day, cells were co-transfected with Keap1–12fl–mCherry and superfolder GFP (sfGFP)–Nrf2 plasmids (Dikovskaya et al., 2019) using Lipofectamine 2000, in phenol-free DMEM supplemented with 10% heat-inactivated FBS. Live cells were imaged the day after transfection before and 1 hour after exposure to PRL-295 (50 μM) or vehicle (0.1% DMSO), using Zeiss LSM 710 confocal microscope operated by Zen software (Zeiss) equipped with 37°C dark environmental chamber supplemented with a humidified CO_2_ source adjusted to 5%. Time-domain fluorescence lifetime imaging (FLIM) was performed on attached SPC-150 SimpleTau module (Becker&Hickl) with Time-Correlated Single Photon Counting capabilities, operated by SPCM software (Becker&Hickl), using tunable InTune laser with 40 MHz pulse frequency at wavelength 490 nm. Image acquisition, processing and analysis using the R-based app FLIM DAtaSet Tool (FLIMDAST) were carried out as described (Dikovskaya and Dinkova-Kostova, 2020). Briefly, the SPCM files were analysed using single-exponential fitting within SPCImage (Becker&Hickle) to obtain the values of fluorescence lifetimes in each pixel of the image and exported as two datafiles containing either photon counts or fluorescence lifetimes in each pixel position, respectively. Photon counts-containing files were further processed in ImageJ, to set all values of pixels outside the specified regions (entire cell, nucleus or cytoplasm) to zero. These modified files were combined with fluorescence lifetime-containing export files within FLIMDAST (https://github.com/DinaDikovskaya/FLIMDAST), where the data acquired from the same cells before and after treatment were assembled into the reference - non-reference pairs to quantify the treatment-induced intensity-independent changes in fluorescence lifetimes and to generate plots of fluorescence lifetimes against pixel intensities. The mean photon numbers within each cellular region quantified in FLIMDAST were exported to calculate the changes in fraction of nuclear GFP fluorescence intensity in cells upon treatment. Confocal images were acquired using 488-nm argon laser (for sfGFP) and InTune laser at 594 nm (for mCherry), immediately before FLIM acquisition.

#### NQO1 enzyme activity assay

Concentration dependence and inducer potency were determined by use of a quantitative NQO1 bioassay (Fahey et al., 2004). Hepa1c1c7 cells (1 × 10^4^ per well) were seeded in 96-well plates, grown for 24 hours, and then exposed in 8 replicates to serial dilutions of PRL-295 or vehicle (0.1% DMSO) for a further 48 hours. Cells were then lysed in digitonin (0.8 g/L in 2 mmol/L EDTA, pH 7.8), and the enzyme activity of NQO1 was quantified in cell lysates using menadione as a substrate. Aliquots of the same cell lysates were transferred to new parallel 96-well plates, and protein concentrations were measured by the bicinchoninic acid (BCA) assay (Thermo Scientific). Inducer potency was expressed as a CD value, i.e. the Concentration that Doubles the NQO1 specific enzyme activity.

#### Animal treatments

PRL-295 was administered to the animals by oral gavage, 4 times, 24 hours apart. Stock solutions in DMSO were prepared and diluted in corn oil to achieve the compound doses indicated in the text and figure legends. The control mice received equal volumes of vehicle (5% DMSO in corn oil). The animals (n = 3–4) were euthanized 3 hours after the administration of the last dose, and tissues (liver, brain, lungs, kidneys and colon) were harvested, washed with PBS, rapidly frozen in liquid N_2_, and stored at −80°C until analysis. For the acute hepatotoxicity experiment, 8-week-old male mice (n = 10) were treated with vehicle of PRL-295 (25 mg/kg body weight) by oral gavage, 3 times, 24 hours apart. After overnight food withdrawal, APAP (300 mg/kg body weight) was dissolved in saline and intraperitoneally administered to the experimental mice, and food was again made available to the animals. The animals were euthanized 24 hours after APAP dosing, and blood was collected. Plasma alanine aminotransferase (ALT) activity was determined using FUJI DRI-CHEM 7000V (FUJIFILM) as a marker of APAP-induced hepatocyte injury. Because of the high inter-individual variation, two outliers (those with the highest and the lowest ALT values) were excluded from each group, leaving an n = 8 for the statistical analysis.

#### Real-time quantitative PCR

Total RNA was extracted from mouse tissues using RNeasy Kit (Qiagen Ltd.). Omniscript RT Kit (Qiagen Ltd.) was then used to reverse-transcribe 500 ng of total RNA into cDNA. Real-time PCR was carried out on Applied Biosystems QuantStudio™ 5 Real-Time PCR System. The TaqMan data for the mRNA for mouse Nqo1 were normalized using mouse Beta-2-Microglobulin (B2M) as an internal control, and the Ces1g data were normalized using hypoxanthine-guanine phosphoribosyltransferase 1 (Hprt1) as an internal control. The TaqMan^TM^ Gene Expression Assay IDs (Thermo) were: Mm00437762_m1 (for B2M), Mm01253561_m1 (for Nqo1), Mm03024075_m1 (for Hprt1), and Mm00491334_m1 (for Ces1g).

#### Immunoblotting

To determine the protein levels of Nrf2, lysates of HL-60 cells were lysed in the SDS lysis buffer containing 50 mM Tris pH 6.8, 2% SDS (w/v) and 10% glycerol (v/v) sonicated for 20 seconds at 20% amplitude using Vibra-Cell ultrasonic processor (Sonic). Protein concentrations were determined by the bicinchoninic acid (BCA) assay (Thermo). Once the samples were adjusted to the same concentration, the sample loading buffer (50 mM Tris pH 6.8, 2% SDS (w/v) and 10% glycerol (v/v) and 0.1% bromophenol blue (w/v)) was added to the samples at a volume of ten percent of the total volume of the lysates. Subsequently, the protein lysates were reduced with 2-mercaptoethanol by adding a volume of five percent of the total volume of the lysate, vortexed briefly to mix and incubated at room temperature for 30 minutes. Proteins were resolved by electrophoresis using 8% Tris-Glycine gels. To determine the protein levels of NQO1, frozen liver tissues were pulverised under liquid nitrogen using a mortar and pestle, and the resulting powder (15 mg) was homogenized in 10 volumes of ice-cold RIPA buffer (50 mM Tris-HCl, pH 7.5, 150 mM NaCl, 1% NP-40, 0.1% SDS, 1% sodium deoxycholate), supplemented with EDTA-free protease inhibitors cocktail (Roche). The insoluble material was removed by centrifugation for 30 minutes at 17,000 x*g* at 4°C. Protein concentration of the supernatant was determined by the BCA assay (Thermo), and adjusted using RIPA buffer with protease inhibitors to the same protein concentration in all samples. The protein lysates were subsequently mixed with 4X LDS loading buffer at a ratio of 3:1. The protein samples were reduced with 2-mercaptoethanol (added to 5 percent of the total volume of the lysate) at room temperature for 30 minutes and the samples were incubated at 70°C for 10 minutes prior to electrophoresis using pre-cast 4–12% gradient NuPage™ gels (Life Technologies) and run with MOPS buffer. The resolved proteins were transferred onto premium 0.45-μm nitrocellulose membranes (Amersham Biosciences). Membranes were blocked in 5% milk-PBST for 1 hour, on a rocker (60–70 rpm), at room temperature, and then incubated with the primary antibodies at 4°C on a rocker overnight. The following antibodies were used: rabbit anti-Nrf2, 1:1000 (Cell Signaling Technology, CST); rabbit anti-NQO1, 1:1000 (CST); mouse monoclonal anti-α-tubulin, 1:5000 (CST); mouse monoclonal anti-β-actin, 1:10,000, Sigma. Subsequently, the blots were washed thrice with PBST for 30 minutes and incubated with secondary antibodies for 1 hour with goat anti-rabbit or mouse 680/800 IRDye (LI-COR) (1:20,000) or goat anti-rabbit HRP (1:5000) (CST) for 1 hour at RT protected from light. The blots were scanned using the Odessey CLx imager using the ImageStudio software (LI-COR) or developed using chemiluminescence (Thermo Fisher Scientific) and captured on an X-ray film (Amersham).

### Quantification and statistical analysis

All quantitative data (except the data in Figure 3) are represented graphically and in the text as mean values ± 1 standard deviation (SD) or standard error of means (SEM), as indicated in the figure legends. The box plots in Figures 3B, 3D, and 3E outline the data range between the 25th and 75th percentiles, with the thick line within the box showing the median, and the whiskers indicating the minimum and the maximum values, excluding the outliers. Student's t-test was used to test for statistical significance.

## Data Availability

Data reported in this paper will be shared by the lead contact upon request. This paper does not report original code. Any additional information required to reanalyze the data reported in this paper is available from the lead contact upon request.
